# Proteomics, Holm Oak (*Quercus ilex L.*) and Other Recalcitrant and Orphan Forest Tree Species: How do They See Each Other?

**DOI:** 10.3390/ijms20030692

**Published:** 2019-02-06

**Authors:** María-Dolores Rey, María Ángeles Castillejo, Rosa Sánchez-Lucas, Victor M. Guerrero-Sanchez, Cristina López-Hidalgo, Cristina Romero-Rodríguez, José Valero-Galván, Besma Sghaier-Hammami, Lyudmila Simova-Stoilova, Sira Echevarría-Zomeño, Inmaculada Jorge, Isabel Gómez-Gálvez, María Eugenia Papa, Kamilla Carvalho, Luis E. Rodríguez de Francisco, Ana María Maldonado-Alconada, Luis Valledor, Jesús V. Jorrín-Novo

**Affiliations:** 1 Department of Biochemistry and Molecular Biology, Agrifood Campus of International Excellence, University of Cordoba, Carretera Nacional IV, km 396, 14014 Córdoba, Spain; b52resam@uco.es (M.-D.R.); bb2casam@uco.es (M.Á.C.); g82salur@uco.es (R.S.-L.); b12gusav@uco.es (V.M.G.-S.); n12lohic@uco.es (C.L.-H.); sghaierbesma@yahoo.fr (B.S.-H.); sira@biognosys.com (S.E.-Z.); isa.m.goga@gmail.com (I.G.-G.); mepv33@gmail.com (M.E.P.); carvalho.kmll@gmail.com (K.C.); bb2maala@uco.es (A.M.M.-A.); 2 Departamento de Fitoquímica, Dirección de Investigación de la Facultad de Ciencias Químicas de la Universidad Nacional de Asunción, Asunción 1001-1925, Paraguay; mromero@rec.una.py; 3 Department of Chemical and Biological Science, Biomedicine Science Institute, Autonomous University of Ciudad Juárez, Anillo Envolvente del Pronaf y Estocolmo s/n, Ciudad Juarez 32310, Mexico; jose.valero@uacj.mx; 4 Plant Molecular Biology Department, Institute of Plant Physiology and Genetics, Bulgarian Academy of Sciences, Acad. G. Bonchev Str. Bl 21, 1113 Sofia, Bulgaria; lsimova@mail.bg; 5 Department of Vascular Biology and Inflammation (BVI), Spanish National Centre for Cardiovascular Research, Melchor Fernández Almagro 3, 28029 Madrid, Spain; inmaculada.jorge@cnic.es; 6 Laboratorio de Biología, Instituto Tecnológico de Santo Domingo, República Dominicana; luisrod95@gmail.com; 7 Department of Organisms and Systems Biology and University Institute of Biotechnology (IUBA), University of Oviedo, Santiago Gascón Building, 2nd Floor (Office 2.9), 33006 Oviedo, Spain; valledorluis@uniovi.es

**Keywords:** holm oak, *Quercus ilex*, 2-DE proteomics, shotgun proteomics, non-orthodox seed, population variability, stresses responses

## Abstract

Proteomics has had a big impact on plant biology, considered as a valuable tool for several forest species, such as *Quercus*, *Pines*, *Poplars*, and *Eucalyptus*. This review assesses the potential and limitations of the proteomics approaches and is focused on *Quercus ilex* as a model species and other forest tree species. Proteomics has been used with *Q. ilex* since 2003 with the main aim of examining natural variability, developmental processes, and responses to biotic and abiotic stresses as in other species of the genus *Quercus* or *Pinus*. As with the progress in techniques in proteomics in other plant species, the research in *Q. ilex* moved from 2-DE based strategy to the latest gel-free shotgun workflows. Experimental design, protein extraction, mass spectrometric analysis, confidence levels of qualitative and quantitative proteomics data, and their interpretation are a true challenge with relation to forest tree species due to their extreme orphan and recalcitrant (non-orthodox) nature. Implementing a systems biology approach, it is time to validate proteomics data using complementary techniques and integrate it with the -omics and classical approaches. The full potential of the protein field in plant research is quite far from being entirely exploited. However, despite the methodological limitations present in proteomics, there is no doubt that this discipline has contributed to deeper knowledge of plant biology and, currently, is increasingly employed for translational purposes.

## 1. Introduction

*Quercus ilex* is the dominant tree species in natural forest ecosystems over large areas of the Western Mediterranean Basin, as well as in the agrosilvopastoral Spanish “dehesa”, with relevance from an environmental, economic, and social point of view [1,2,3]. These ecosystems are currently subjected to different threats including: very old individuals, overexploitation and poor regeneration, inappropriate livestock management, and the severe effect of forest decline attributed to fungal attack (such as *Hypoxylon mediterraneum* or *Phytophthora cinnamomi*), extreme temperatures and extended drought periods, among other factors [3,4,5]. This already worrisome situation could become even worse under the threat of the foreseen climate change scenario [6,7]. In order to preserve such an invaluable ecosystem, these problems must be faced, and biotechnology is a valid alternative that could contribute to resolving some of these problems. However, the development of biotechnological approaches for the conservation, sustainable management and regeneration of *Q. ilex*, and other forest ecosystems is hampered by the limited knowledge of their biology, especially at the molecular level. Biochemical and molecular biology research is a priority for designing biotechnological approaches for simultaneously conserving and exploiting forest ecosystems. Plausible, realistic, and impactful first steps to ameliorate this situation could include the characterization of its biodiversity and the selection of elite genotypes based on molecular markers. In this context, protein profiling through different proteomic approaches would be highly useful [8].

Since 2003, our research group has worked on the proteomics of forest tree species, with a first publication in 2005 [9]. Our investigations have been focused mostly on *Quercus ilex* subsp. *ballota* [Desf.] Samp., and, to a lesser extent, on various *Pinus* spp., including *P. radiata* [10], *P. occidentalis* [11], *P. halepensis* [12], and *P. pinea* [13]. All these forest tree species can be classified and catalogued as orphan due to the absence of molecular studies and, depending on their seed characteristics, properties, and maturation, as highly recalcitrant (non-orthodox) plant systems [8], because unlike orthodox seeds, non-orthodox seeds are damaged by loss of water and are unstorable for practical purposes.

**Figure 1:**
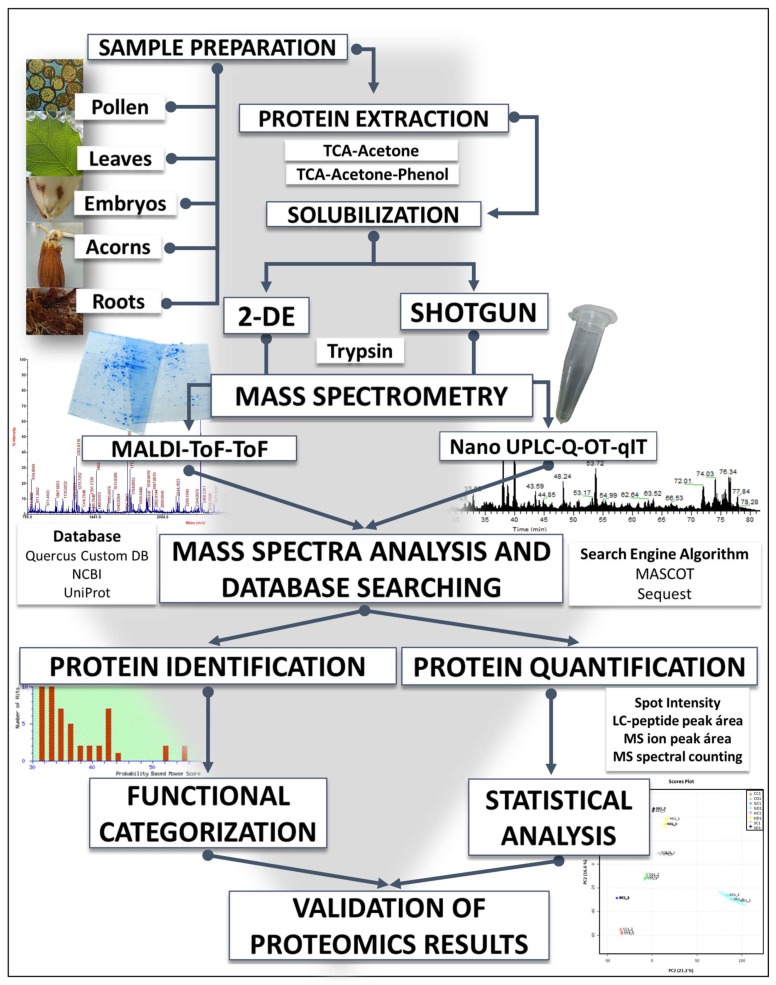
Workflow of a proteomics experiment, from sample preparation to data analysis and validation. It includes alternative, complementary approaches or strategies, based on MS analysis of proteins (top-down) or tryptic peptides (bottom-up), either gel-based or gel-free. LC: liquid chromatography; MS: mass spectrometry.

So far, our proteomics-based research on *Q. ilex* has focused on descriptive and comparative proteomics sub-areas (Figure 1). In addition, we have begun to explore the field of posttranslational protein modifications, specifically phosphorylation [14]. Using 2-DE based strategy coupled with mass spectrometry (MS), and, to a lesser extent, shotgun approaches, the proteome of seeds, pollen, roots, and leaves, both in adult plants and plantlets, have been partially characterized, and differences in protein profiles among provenances have been identified [9,15,16,17]. In an attempt to study the non-orthodox character of the species, we have further investigated the proteome of mature acorns, as well as the differences between developmental stages of seed maturation and germination [18,19]. Furthermore, our research has been focused on studying plant responses to abiotic and biotic stresses related to decline syndrome, mainly drought and *P. cinnamomi* infestation, as well as differences among *Q. ilex* provenances from Andalusia combining proteomics, morphometry, and physiological analysis [17,20,21,22,23]. This manuscript does not intend to be a review of the field of proteomics, because there are already a high number of publications available in the literature [24,25,26,27,28,29,30,31,32], or discuss terminology or scientific standards mandated by the corresponding Minimum Information about Proteomics Experiment (MIAPE) guidelines [33]. On the other hand, the application of proteomics in forest tree research has also been the subject of some previous reviews, with a quite descriptive point of view [34,35]. So, in this review, we intend to emphasize all the lessons learnt through fifteen years working on *Q. ilex*, which includes everything from the experimental design, protein extract preparation, MS analysis, confident identification and quantification of protein species to data interpretation from a biological perspective [36,37]. For most of the mentioned issues, all the studies carried out on an orphan and extremely recalcitrant experimental system such as *Q. ilex* have been highly challenging. Proteomics is more than only a single table of possible protein identifications, i.e., database matches, or, even in the best of cases, ortholog identifications and their technical validation. Literature, including our own publications, may contain errors, speculations, and incorrect interpretations, which are waiting to be revised. 

## 2. How *Quercus ilex* Is Seen by Proteomics

### 2.1. ‘Only a Small Percentage of the Total Protein Is Extracted and Solubilized, So We Deal with the Extractome Rather Than with the Real Proteome’

There are two major approaches for making protein extracts, independently of the subcellular compartment, based on either precipitation or solubilization. Both approaches are the most common protocols to extract proteins and these should be optimized in each organism. In our hands, precipitation methods have always given the best results in terms of protein yield as determined by colorimetric methods, generally using Bradford assay (20 mg g^−1^ fresh weight from *Q. ilex* leaf, as an example) [17]. Depending on the chemical composition and protein content of the organ analyzed and the amount of tissue available, trichloroacetic acid (TCA)-acetone precipitation alone or combined with phenol partitioning, followed by ammonium acetate-methanol precipitation, have consistently yielded the best results [37]. Table 1 collects the main features of the *Q. ilex* publications cited in this review. Protein yield and even recovery across a wide range of proteins is a constant concern in protein biochemistry. Remarkably, the protein concentrations of extracts are commonly absent in many publications, although the protein quantification of the extracts has been expressly stated in the material and methods section.

It is true that absolute quantification by current protocols (Lowry, Bradford, bicinchoninic acid (BCA), amido black) is not always reliable, as up to ten-fold difference may be observed between different protocols. Still, they may be valuable for comparative purposes and reproducibility [42].

We have extracted proteins from different organs of more than 25 different plant species, both woody and herbaceous. Protein content in those extracts was consistently lower than 10% (in the 1-20 mg g^−1^ Dry Weight (DW) range [43]) of the total as determined using the Kjeldahl method [44], with some legume species having the highest values [43]. For the acorns, pollen, and leaves of *Q. ilex*, values of 3–6, 8‒14, and 10–40 mg g^−1^ DW were reported, respectively [16,17,38,45] (Table 1). Even when applying Osborn′s sequential extraction protocol to *Q. ilex* seeds [46], the total protein content obtained was around 15 mg g^−1^ DW as determined using Bradford assay, which represents around 30% of the total protein as determined using near-infrared spectroscopy (NIRS) [47]. These data lead us to estimate mistakes and make speculations while interpreting our proteomics data from a biological point of view, as we are clearly not recovering and therefore not examining the huge submerged part of the proteome iceberg.

### 2.2. The Plant Proteome is Highly Variable and Therefore Requires Careful Experimental Design

This was one of the first major lessons that we learnt when working with *Q. ilex*. We have observed that the 2-DE protein profile of leaf samples collected from field trees is not reproducible. Only after systematic analysis of the protein pattern obtained, we could show that results strongly depended on leaf position (top, bottom), leaf orientation (north, south, east, west) and sampling time (morning, afternoon, evening) [9]. These observations were more than obvious considering the sessile and plastic nature of plants, but they were not considered when the experiments were designed. The average value of the coefficient of biological variance (CV) for protein abundance (spot intensity) was found close to 60 % for field samples and close to 45% for plantlets grown under controlled conditions, while values of 20‒25 % were found for analytical variability [9,15]. The average standard error of spot intensity decreased by a factor of two when the number of biological replicates increased from two to twelve (from an average of 120 to 60 ng protein per spot) [9,15]. High variability is a common feature for plants. Plant organs are complex mixtures of tissue and cell types, each with their own protein signature. In addition, individuals of non-domesticated plants exhibit high variability. Because of these issues, a significant number of biological replicates should be considered to decrease the effect of variability in our results. The direct consequence of this is the need to characterize the variability beforehand using test measurements and then perform an exhaustive analysis to determine the number of required replicates. Alternatively, the analytical approach may have to be refined. Due to obvious limitations (space, time, equipment, and costs), it is not always possible to perform experiments based on a large number of replicates. However, the actual concern is how the data are interpreted. For comparative purposes, we only consider as variable spots those that are consistent (present in all the replicates), and with lower CVs than the average of the sample [9,48]. A higher ratio between samples makes more confident those quantitative differences observed, although sometimes only qualitative differences may be trusted. All these issues, together with tips to be considered for proper experimental designs and statistical tests (mostly multivariate and clustering), should be contemplated when a 2-DE based proteomics experiment is planned. Moreover, the correct analysis and interpretation of the data should be contemplated, thus, both are discussed in more detail in this review [36,48].

Generally, the proteome is discussed as a sum of the individual proteins identified and analyzed using a univariate approach, such as ANOVA, instead of being considered globally as a part of a biological entity and analyzed using a multivariate approach. Since univariate approaches are negatively affected by the raw structure of the data, they do not detect trends or groups increasing the false positives. On the other hand, multivariate analyses such as principal component analysis (PCA), partial least squares (PLS), principal coordinate analysis (PCoA), or partial least squares-discriminant analysis (PLS-DA), should be employed because they describe trends and reduce the complexity of the data [49]. Despite these multivariate approaches being intended to reduce data dimensionality, PCA seeks a few linear combinations of variables that can be used to summarize data while PLS considers how each predictive variable may be related to the dependent variable [49]. In any case, the combination of both univariate and multivariate approaches that provide a comprehensive overview of the data with single protein analyses and multiprotein tendency maximize the information obtained from the datasets [36].

### 2.3. Only a Small Fraction of the Present Protein Species Is Visualized and Identified by Any Given Approach

**Figure 2:**
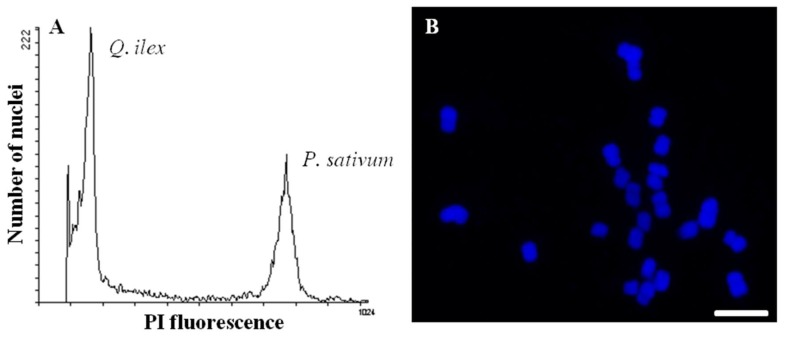
(**A**) Uniparametric histograms of fluorescence intensities of the nuclei of *Q. ilex* and *Pisum sativum*, used as a control, after staining with propidium iodide (PI). The 2C nuclear DNA content of *P. sativum* is 9.09 pg. (**B**) Somatic chromosomes in root tip cells of *Q. ilex*. Scale bar = 10 µm.

The number of spots resolved in different *Q. ilex* samples subjected to 2-DE analysis was in the range of 200‒600 spots, depending on organ of the plant (seed, pollen, or leaf), range of isoelectric focusing (IEF) pH (5‒8 as a general strategy), and staining protocol. Of the total spots subjected to mass spectrometry less than 50% of hits could be identified, depending on the database used (see above section on protein identification, Table 1) [15,16,17,18,20,21,22,45,47]. However, assuming the possibility of spot comigration, the maximum number of resolved proteins is below 1000. This amount of protein is notably increased into the thousands when a nLC-ESI-MS/MS shotgun approach is employed. Thus, up to 4500 peptides could be resolved in germinating seeds through LC-MS/MS shotgun analysis [19]. Assuming a theoretical calculation based on 3 peptides per protein, around 1650 protein species could be resolved. Thus, the use of a shotgun approach and a huge growth in bioinformatics has led to an explosion of data in the field of proteomics. Nevertheless, although the integration of both approaches is expanding their application in the identification of a higher number of peptides, their focus and strengths remain in the analysis of DNA sequences and genomes of plant species. The sequencing of the *Q. ilex* genome, which is indeed one of our next objectives, would be considered as a final step to integrate all the proteomics data obtained so far. However, this issue can currently be solved using the recently published genomic data available for other species of the genus *Quercus,* such as. *Q. robur* [50], *Q. lobata* [51], and *Q. suber* [52]. The genome of *Q. robur* has an estimated size of 740 Mb/C [53] and consists of 17,910 scaffolds, of length 2 kb or longer, with a total length of 1.3 Gb [50]. On the other hand, the first draft of the genome of *Q. lobata* has a genome size of approximately 730 Mb/C and 18 512 scaffolds (> 2 kb) [51]. A comparison of nuclear sequences between both *Quercus* species indicated 93% similarity [51]. Lesur et al. [54] have reported the most comprehensive transcript catalog assembled to date for the genus *Quercus*, with 91,000 annotated contigs. With the aim of sequencing the *Q. ilex* genome, our group has started to address basic aspects of the genome, such as estimation of the nuclear DNA content and the number of chromosomes of *Q. ilex.* The estimated genome size was approximately 930 Mb/C with a total length of 1.87 Gb, as assessed using flow cytometry [55] (Figure 2A). Zoldos et al. [56] and Chen et al. [57], using the same methodology as with *Q. ilex*, reported a higher *Q. robur* genome size than the data reported in Plomión et al. [50] (approximately 914 Mb/C and 890 Mb/C, respectively). Previous cytological studies established that the number of chromosomes in the genus *Quercus* has remained stable over time, being mainly 2*n* = 24. Cytogenetic methods were used for chromosome count in root tip squashes of *Q. ilex* [58]. As expected, *Q. ilex* had the same chromosome number as *Quercus* spp. (Figure 2B). All chromosomes are quite similar morphologically, so that other cytogenetic methods should be used to identify all the chromosomes individually.

The proteome data can also be complemented using a transcriptomic approach. The first *de novo* assembled transcriptome of the non-conventional plant *Q. ilex* has recently been published [39,40,59]. The transcriptome of a mixture of different tissues of *Q. ilex* using two sequencing platforms, Illumina and Ion Torrent, and three different algorithms, MIRA, RAY, and TRINITY, was analyzed. Firstly, around 62,628 transcripts were identified using the Illumina platform (Illumina HiSeq 2500) [39]. Then, in a revised version of the *de novo* assembled transcriptome, the Ion Torrent sequencing platform was used, and 74,058 transcripts were identified [59]. The data reported for *Q. robur* and *Q. lobata* genomes and for the *Q. ilex* transcriptome express at least one order of magnitude higher than the number of expressed, visualized, and identified protein species in 2-DE or shotgun observed in our experiments—even without considering possible posttranslational modifications (PTMs)—although the non-consolidated nature of our data is considered. With these values in mind, we should only deal with a minimum fraction of the total proteome and any biological interpretation of the data should be made with caution, being as conservative as possible and avoiding speculations, especially if data are not validated.

The integration of omics approaches (genomics, transcriptomics, proteomics, and metabolomics) are commonly used to further our knowledge about plant biology. The data identified in each approach is quite variable, which depends on the available databases. For example, a total of 62,629 transcripts, 2380 protein species, and 62 metabolites were recently described in *Q. ilex* [39]. In spite of having a considerably lower number of proteins and metabolites than transcripts, proteomics and metabolomics could give a more connected understanding of the phenotype of the plant species. Thus, the integration of multi-omics studies with phenotypic and physiological data in the systems biology direction are necessary to obtain a better understanding of the molecular mechanisms underlying phenotypes of interest.

### 2.4. Gene Product Identification? Or Just Hits or Matches to Orthologs?

Proteome analysis of *Q. ilex* has been prevented for a long time due to the almost total absence of DNA or protein sequence entries in the available databases and, possibly, errors in the deposited sequences themselves. Consequently, protein identification from MS data usually had low peptide-to-spectra matching, even using *de novo* sequencing and sequence similarity searching (i.e., [9,15]). The concern that proteomics was only possible with organisms whose genome are properly sequenced and annotated, was a recurrent matter of discussion with Dr. Juan Pablo Albar (1953-2014, R.I.P.). Even considering that the possibility of orthologs identification already provided useful information on mechanisms and metabolism in many cases, some issues remained unresolved. In parallel, plant breeding programs request increasingly accurate gene information rather than just the ortholog approximation. For this reason, we changed our strategy and decided to build a custom *Quercus* protein sequence database to improve the success rate of peptide and protein identifications and assignments [41]. This database is continuously updated and allows successful reviewing of existing data sets for the scientific community. The latest version of our custom *Q. ilex* database contained 3541 annotated proteins from the Ion Torrent platform [59]. At this moment, the number and confidence of the identifications can be carried out using the presence of whole genome sequencing of several forest tree species [60]. However, despite admitting positive identification (matches in some cases), the confidence value is not the same for all the proteins, although we assigned them the same probabilistic value when the data were interpreted from a biological point of view. Thus, the shotgun strategy in the proteome analysis of a pool of tissues (embryo, cotyledon, leaves, and root) from *Q. ilex* resulted in 7000 peptides and 1600 putative protein identifications when the species-specific database created from the *Q. ilex* transcriptome was used [40]. The confidence values obtained in this study was in the range 1‒35 peptides per protein, 1‒93 % sequence coverage, and 1‒335 score values (using SEQUEST algorithm) [61]. However, almost 50% of identifications showed at least one parameter of low confidence (1 peptide per protein, sequence coverage <10%, or score value <2). These issues, although relevant, were rarely discussed openly, as blind acceptance of the results provided by the matching algorithm was in many cases easier and considered enough. However, publication of a list of sequence assignments is no longer enough to justify it. In the case of orphan species, ortholog identification does also not resolve the doubts about what protein species (different products of the same gene), isoforms, or allelic variants are present in a biological system nor indicate what they signify. If the aim is to obtain biological understanding of the data beyond description, proteomics data must be validated, especially in the case of orphan species; otherwise it remains largely speculative. 

### 2.5. Methods and Protocols Must Be Validated and Optimized for Each Experimental System

The final goal of a proteomics experiment is to identify, characterize, and quantify as many protein species as possible. Different workflows, protocols, technology platforms, and algorithms are available, each one with its own signature and characteristics [27]. Small variations in a protocol used, such as different gel stains, may result in a different partial view of the protein ‘firmament’. In our experience with different biological systems, including plants, bacteria, yeast, fungi, and animal cells [27,62,63], each protocol should be optimized for the experimental system under investigation, due to the presence of polysaccharides, phenolics, nucleic acids, salts, and other small metabolites in each biological sample.

Biologists are often far away from an analytical chemist’s orthodox thinking, and this sometimes leads us to commit important errors in our biological interpretation of analytical results. It is of paramount importance to understand the properties of the analytical techniques employed, including selectivity, precision, accuracy, recovery, linearity range, limit of detection and quantification, robustness, and stability. Both the linearity and the limit of detection, outside of their working range, are of special relevance considering that the comparisons are not valid. This is equally applied to 2-DE and shotgun approaches [41,61,64,65,66]. Nevertheless, the output of analytical proteomics workflows should never be taken at face value, but they must be validated and corroborated for each experimental system. Both for 2-DE and shotgun, we usually perform a calibration curve based on different dilutions of a sample; from these serial dilution assays and depending on the protein concentration of the sample, we will see how many proteins are identified (major and minor proteins) and how many are confidently identified, proven using similar ratios in dilution and protein or peptide amount [41,61,64,65,66].

### 2.6. 2-DE and Shotgun Platforms Are Complementary

Roughly up to the year 2000, 2-DE based workflows were the predominant platforms employed in plant proteome analysis, and since then, analytical technology has been progressing to second (isotopic or isobaric labelling) and third generation (shotgun, gel-free label-free) approaches, with the latter nowadays being dominant [26]. Considered as an obsolete technique by some scientists, 2-DE based workflows are still valid for some purposes such as top-down proteomics and the identification of protein species or proteoforms of the same gene [32,67]. In our investigations on *Q. ilex*, we have followed the same tendency. The choice of one or other strategy depends on different factors, such as equipment availability, expertise, technical skills, and cost, among others. It is outside the scope of this paper to discuss the potential and limitations of the different techniques; for that, we refer the reader to previously published literature [24,27,30]. Usually, thousands of proteins are identified using a shotgun approach versus hundreds when using a 2-DE based strategy (Table 1). However, both approaches are complementary as the number of common proteins identified using each approach is not always high. Thus, we have used both approaches in parallel (2-DE/MALDI-TOF/TOF, and nLC-ESI-LTQ Orbitrap) in the analysis of seed extracts at different times after germination [19]. The *Quercus*_DB protein database [41], combined with UniProtKB/TrEMBL, UniProtKB/SwissPrto and NCBInr databases, the taxonomy restriction to *Viridiplantae*, and the SEQUEST algorithm were used. A total of 540 consistent spots were resolved using 2-DE in the 5‒8 pH range. Out of the 103 variable spots subjected to MALDI-TOF/TOF analysis, 90 were identified [19]. On the other hand, up to 1650 protein species were identified using nLC/MSMS, with 25% of them not annotated. Both proteomics approaches (gel-based and shotgun) were complementary, with shotgun increasing the coverage of the proteome analyzed by over two-fold, and both providing similar results and supporting the same conclusions on the metabolic switch experienced by the seed upon germination [19]. The highest number of matches was obtained when 1-D SDS-PAGE was combined with nLC/Orbitrap/MS (Q- Exactive), with up to 9000 peptides and 1800 proteins identified at an estimated 1 % FDR from a *Q. ilex* extract obtained from a mixture of organs (seeds, leaves, roots, and pollen) [65]. The number of identified proteins depended on the algorithm (Mascot, ProteinPilot, and Maxquant) and database (NCBInr with restrictions to Viridiplantae, Fabids, Rosids, or *Quercus*) [65].

### 2.7. How Proteomics Sees Quercus ilex

Proteomics has been a helpful approach for our current research projects with *Q. ilex*, both from a basic research and from a translational point of view. Below, we will briefly summarize what contributions have been made with references to original articles for deeper discussion.

### 2.8. Characterizing Biodiversity

One of our first objectives was to characterize and catalog Andalusian *Q. ilex* populations and provenances based on the leaf 2-DE profile, using field and greenhouse samples [9,15]. Due to the high variability existing in this species, we failed with the leaf proteome, so we decided to analyze different plant tissues with a more stable proteome, such as seed and pollen. Protein extracts from these tissues were subjected to 1-DE (SDS-PAGE) or 2-DE (IEF/SDS-PAGE) protein separation, and variable bands or spots among the provenances were analyzed using MALDI-TOF/TOF MS after tryptic digestion [16,68,69]. In seed extracts, 1-DE data allowed the grouping of populations defined by their geographical location (North, South, East, West) and climate conditions (mesic and xeric). Thus, acorn flour extracts from the most distant populations were analyzed using 2-DE, and 56 differential spots were proposed as markers of variability (Table 1) [16]. A comparison of 1-DE and 2-DE protein profiles of pollen extracts from four provenances in Andalusia revealed significant differences, both qualitative and quantitative (18 bands and 16 spots, respectively), with most of them related to metabolism, defense/stress processes, and cytoskeleton [69]. Similar results have been found when triploid and tetraploid *Populus deltoids* pollen were compared [70]. 

A multivariate statistical analysis carried out on bands and spots clearly showed distinct associations between provenances, which highlighted their geographical origins. Other complementary approaches, including morphometric, NIRS, and microsatellite analysis, have been used for cataloguing *Q. ilex* populations, with good agreements between the different techniques [16,38,45,69,71].

### 2.9. Adaptation to Biotic and Abiotic Stresses

Responses to biotic and abiotic stresses are considered as the most covered topic in plant research, in general, and forest tree research, in particular. For instance, nutritional deficiency studies have been approached using proteomics in *Fagus sylvatica* and *P. massoniana* [72,73], oxidative stress in *Populus simonii x P. nigra* [74], salt in *Robinia pseudoacacia* and *Paulownia fortune* [75,76], drought in *Platycladus orientalis* [77], *P. halepensis* and *Larix olgensis* [78,79], UV light in *P. cathayana*, and *P. radiata* [80,81,82], heavy metals in *P. yunnanensis* [83], and pathogens in *P. tomentosa* [84]. *Quercus ilex* responses to abiotic (drought) and biotic (*P. cinnamomi)* stresses and the variability in such response among populations are a key objective of our research, ultimately aimed at characterizing and selecting elite genotypes with high levels of tolerance and resistance to both stresses, conferring fitness advantages in a climate change scenario.

For that purpose, changes in the leaf protein profile occurring in drought stressed or fungal inoculated plants were analyzed using 1-DE and 2-DE coupled twith MALDI-TOF/TOF MS [15,17,20,21,69]. The resulting proteomics data were correlated with drought tolerance, plantlet growth, presence of toxicity symptoms, and physiological (water regime and photosynthesis) parameters.

Plantlets from seven *Q. ilex* provenances distributed all over the Andalusian geography showed different levels of tolerance to drought as well as differential changes in their 1-DE and 2-DE protein profiles upon water withholding [21]. Variable spots in leaf extracts from the most contrasting populations in terms of drought tolerance were subjected to 2-DE MALDI-TOF/TOF MS analysis, resulting in 28 consistent spots varying in abundance, with 18 unique protein species identified (Table 1) [21]. A general tendency of reduction in protein abundance, especially in proteins related to ATP synthesis and photosynthesis, was observed upon water withholding. The most dramatic decrease was observed in the less tolerant seedling population [21]. The same trend was observed in sunflower plants subjected to drought stress [85]. Upon water availability reduction, changes in the protein profile were observed in two sunflower genotypes, a susceptible and a tolerant one. Two genotype-dependent, and 23 (susceptible genotype) and 5 (tolerant genotype) stress-responsive variable proteins were identified. A general decrease in enzymes of the photosynthesis and carbohydrate metabolism was observed in the susceptible genotype, suggesting inhibition of energetic metabolism. Such changes were not observed in the tolerant genotype, indicating a normal metabolism under drought stress [85].

In a similar study, responses to the fungal pathogen *Phytophthora cinnamomi*, one of the agents that triggers the decline syndrome in *Quercus* spp., were studied by our research group using one-year old seedlings from two Andalusian provenances with different levels of susceptibility [17]. Leaf protein profiles were analyzed in non-inoculated and inoculated seedlings using a 2-DE coupled with MS proteomics strategy. Seventy-nine protein species that changed in abundance upon inoculation were identified after MALDI-TOF/TOF analyses (Table 1) [17]. Out of them, 35 were chloroplastic, with 7 being a part of the photosynthetic electron transport chain and ATP synthesis, 19 belonged to the Calvin cycle and carbohydrate metabolism (with 8 large RubisCO protein spots), and 10 involved in other carbon and nitrogen pathways [17]. A general decrease in protein abundance was observed, being less pronounced in the least susceptible provenance [17]. The same trend clearly manifested in their photosynthesis, amino acid metabolism, and stress/defense proteins. On the contrary, some proteins related to starch biosynthesis, glycolysis, and stress related peroxiredoxin showed an increase upon inoculation [17]. These changes in protein abundance correlated with the estimated physiological parameters and were frequently observed in plants subjected to drought stress [17].

### 2.10. Development: Seed Maturation and Germination

Last but not least, proteomics has been employed to analyze the proteome of seeds and changes associated to seed maturation and germination in an attempt to characterize and differentiate, at the molecular level, orthodox and non-orthodox species and zygotic and somatic embryos ([18,19,86,87,88,89,90,91,92,93]; this study is of great importance for propagation and seed conservation programs.

Sghaier-Hammami et al. [18] reported on the 1-DE and 2-DE protein profile of the different parts of the seed: embryonic axis, cotyledons, and tegument. One hundred and ninety variable proteins among the three parts of the seed analyzed were identified using MALDI-TOF/TOF (Table 1). Cotyledon presented the highest number of metabolic and storage proteins (89% of legumins), while the embryonic axis and tegument had the largest number of fate group and defense-/stress-related proteins, respectively. This distribution was in good agreement with the biological role of the tissues and demonstrated a compartmentalization of pathways and a division of metabolic tasks between the embryonic axis, cotyledon, and tegument.

Romero-Rodríguez et al. [19] analyzed changes in the protein profile of *Q. ilex* seeds upon germination using complementary 2-DE coupled with MALDI-TOF/TOF and shotgun nLC-ESI-MS/MS approaches. Proteins from embryos at 0 h and 24 h post imbibition, as well as from shoot seedlings at 1 and 4 cm stages were separated using 2-DE, resulting in a total of 540 spots resolved, 103 of which were changes between developmental stages. Ninety differentially accumulated proteins were identified after MALDI-TOF/TOF analysis (Table 1). Proteins related to energy metabolism and photosynthesis were accumulated during seedling establishment. Few proteins showed quantitative differences during the germination period (0 to 24 h post imbibition). When a gel-free shotgun approach was used, 153 differentially accumulated proteins between non-germinated and germinated seeds were identified. Data suggested that the mature non-orthodox seeds of *Q. ilex* have the mechanisms necessary to ensure the rapid resumption of the metabolic activities required to start the germination process and to *de novo* synthesize the biomolecules required for growth, and this makes a big difference from orthodox seeds [19].

## 3. Conclusions and Perspectives

With this review, we aimed to illustrate the potential and limitations of a proteomics approach applied to non-model forest tree species. These species are considered experimental system that have been quite challenging due to their biological characteristics, recalcitrant nature, and the lack of phenotypic, physiological, or molecular information. The full potential of proteomics has been far from fully exploited in investigations in most plant biology research such as *Q. ilex*. In order to obtain a deeper coverage of the *Q. ilex* proteome, subcellular fractionation techniques or protein depletion and fractionation based on physicochemical or biological properties should be implemented. Apart from proteome subfractionation (e.g., [94]), future research will go in the direction of selected reaction monitoring (SRM), multiple reaction monitoring (MRM), and MS-western or data independent searches based on proteotypic peptides [95]. Some areas of proteomics, such as PTMs and interactomics, have not been approached so far in *Q. ilex* studies, the latter being necessary for understanding the mechanisms that result in a phenotype from the genotype. The lack of an accurate and annotated sequenced genome of *Q. ilex* is an important gap in our research because this is essential for obtaining confident gene product identification and describing protein species or forms as a result of alternative splicing and posttranslational events. Moreover, a sequenced genome would open the door to the application of newly developed approaches such as targeted proteomics.

We have learnt the importance of a proper experimental design and statistical analysis of the data, as well as the relevance of optimizing and validating the techniques employed in each experimental system, plant species, organ, and tissue. We have the possibility of using a range of platforms, methods, and protocols that are complementary, helping us to acquire broader proteome knowledge. In some regards, we may have to broaden our biologist mentality and assume the mindset of an analytical chemist. Plant biologists publishing papers on proteomics should go beyond the blind acceptance of the data provided by the algorithms that come from proteomics services; we should not expect proteomic technicians to be familiar with plant biology. Proteomics by itself may be considered mostly descriptive, and the biological interpretations following, to some extent, as just speculations. Thus, it is necessary to integrate proteomics research with other techniques, including morphometry phenotyping, physiology, classical biochemistry, and other -omics in order to validate the data and procure a more realistic and non-biased view of living organisms [96,97,98,99,100]. It is still astounding how in some publications the whole biology of an organism is discussed and compared with others using data from a poorly designed experiment with a small number of replicates and a minimum fraction of the proteome covered.

Even so, proteomics is making important contributions to the knowledge of living organisms and can be confidently employed for translational purposes. By using proteomics, we have been able to discriminate provenances of *Q. ilex* from Andalusia, find out the differential responses to biotic and abiotic stresses among them, and establish some of the differences existing between orthodox and non-orthodox plant species. New directions in *Q. ilex* research will lead to the identification of allergens in pollen grains and acorns and the characterization of wood materials, which are objectives clearly approached by proteomics [101,102,103].

## Figures and Tables

**Table 1:** Relevant results concerning proteomics research on *Quercus ilex* carried out by our group.

Author	Year	Plant Organ	Protein Yield (mg g^−1^ DW Tissue) ^a^	Proteomic Strategy	Features ^c^	Identified Proteins	Proteome Database ^e^
Jorge [9]	2005	Leaf	Data not reported *; L	2-DE MALDI TOF/TOF	350	20 out of 100 spots	NCBI: restriction to Viridiplantae
Jorge [15]	2006		Data not reported *; L		400	24 out of 100 spots	
Echevarría-Zomeño [20]	2009		7 *; L		390	12 out of 46 spots	SwissProt, trEMBL and NCBI: restriction to Viridiplantae
Valero-Galván [16]	2011	Seed	6 *; B		240	16 out of 56 spots	NCBI: restriction to Viridiplantae
Valero-Galván [38]	2012	Pollen	15 ^§^; B	2-DE MALDI-TOF/TOF	600	77 out of 100 spots	UniProtKB restricted to *Arabidopsis*; Phytozome restricted to *Populus* and *Eucaliptus*; Custom-build database from *Quercus* ESTs ^f^
				Shotgun (nLC-MS/MS) ^b^	Data not reported	273	
Valero-Galván [21]	2013	Leaf	10 ^§^; B	2-DE MALDI-TOF/TOF	230	18 out of 28 spots	NCBI: restriction to Viridiplantae
Sghaier-Hammami [17]	2013		40 ^§^; B		480	80 out of 480 spots	
Simova-Stoilova [22]	2015	Root	3 ^§^; B		360	79 out of 90 spots	NCBI and UniProtKB: restriction to Viridiplantae
Romero-Rodríguez [14]	2015	Embryo	150 ^§^; B		480	20 ^d^ out of 55 spots	NCBI, UniProtKB: restriction to Viridiplantae and Custom *Quercus* database ^f^
Sghaier-Hammami [18]	2016	Cotyledon	2 ^§^; B		440	50 out of 153 spots	NCBI: restriction to Viridiplantae
		Embryo	80 ^§^; B		470	50 out of 153 spots	
		Tegument	0,4 ^§^; B		420	40 out of 153 spots	
López-Hidalgo [39]	2018	Pool of tissues: acorn, embryo, cotyledon, leaf and root	40 ^§^; B	Shotgun (nLC-MS/MS) ^b^	58600	2830	SwissProt: restriction to Viridiplantae/ Custom-build specie database ^f^
Romero-Rodríguez [19]	2018	Seed	25 ^§^; B	2-DE MALDI-TOF/TOF	540	90 out of 103 spots	NCBI, UniProtKB/TrEMBL and UniProtKB/SwissProt restricted to Viridiplantae; Custom-build *Q. ilex* database ^f^
				Shotgun (nLC-MS/MS) ^b^	3113	1650	

^a^ Approximated values have been adjusted to the unit. * = TCA extraction method and § = TCA-Phenol extraction method. Final pellet was resuspended in a solution containing 9 M urea, 4% CHAPS, 0.5% Triton X100, and 100 mM DTT. Proteins were quantified using the Lowry (L) or Bradford (B) protocols; ^b^ The equipment used in the shotgun strategy was nLC-MS/MS (orbitrap, Q-OT-qIT); ^c^ Spots resolved using 2-DE or peptides identified using shotgun LC-MS/MS; ^d^ This value corresponds to identified phosphoproteins; ^e^ MASCOT and SEQUEST search engines were used with MALDI-TOF/TOF and shotgun LC-MS/MS data, respectively; ^f^ The custom-build databases from the genus Quercus and Q. ilex have been published by Guerrero-Sanchez et al. [40] and Romero-Rodríguez et al. [41].
